# *Agrobacterium tumefaciens estC*, Encoding an Enzyme Containing Esterase Activity, Is Regulated by EstR, a Regulator in the MarR Family

**DOI:** 10.1371/journal.pone.0168791

**Published:** 2016-12-30

**Authors:** Surawach Rittiroongrad, Nisanart Charoenlap, Suparat Giengkam, Paiboon Vattanaviboon, Skorn Mongkolsuk

**Affiliations:** 1 Laboratory of Biotechnology, Chulabhorn Research Institute, Bangkok, Thailand; 2 Department of Biotechnology, and Center of Emerging Bacterial Infection, Faculty of Science, Mahidol University, Bangkok, Thailand; 3 Center of Excellence on Environmental Health and Toxicology, Bangkok, Thailand; 4 Program in Applied Biological Sciences: Environmental Health, Chulabhorn Graduate Institute, Bangkok, Thailand; Universidade de Sao Paulo Instituto de Biociencias, BRAZIL

## Abstract

Analysis of the *A*. *tumefaciens* genome revealed *estC*, which encodes an esterase located next to its transcriptional regulator *estR*, a regulator of esterase in the MarR family. Inactivation of *estC* results in a small increase in the resistance to organic hydroperoxides, whereas a high level of expression of *estC* from an expression vector leads to a reduction in the resistance to organic hydroperoxides and menadione. The *estC* gene is transcribed divergently from its regulator, *estR*. Expression analysis showed that only high concentrations of cumene hydroperoxide (CHP, 1 mM) induced expression of both genes in an EstR-dependent manner. The EstR protein acts as a CHP sensor and a transcriptional repressor of both genes. EstR specifically binds to the operator sites OI and OII overlapping the promoter elements of *estC* and *estR*. This binding is responsible for transcription repression of both genes. Exposure to organic hydroperoxide results in oxidation of the sensing cysteine (Cys16) residue of EstR, leading to a release of the oxidized repressor from the operator sites, thereby allowing transcription and high levels of expression of both genes. The *estC* is the first organic hydroperoxide-inducible esterase-encoding gene in alphaproteobacteria.

## Introduction

*Agrobacterium tumefaciens* is a Gram-negative soil bacterium that causes crown gall tumor in a wide variety of dicotyledonous plants worldwide. As a plant pathogen, *A*. *tumefaciens* is exposed to reactive oxygen species (ROS), including H_2_O_2_, superoxide anions, and lipid hydroperoxides that are generated by of active plant defense responses and from other microbes in the environment [1]. Oxidative stress protection systems in *A*. *tumefaciens* have been partially characterized. At least three oxidative stress sensors/transcriptional regulators, SoxR, OxyR, and OhrR that sense increased levels of superoxide anion, H_2_O_2_ and organic hydroperoxides, respectively, have been investigated [2–6]. SoxR directly regulates *sodBII*, which encodes iron containing superoxide dismutase, whereas OxyR controls the expression of the *katA* catalase gene [4–6]. OhrR regulates the expression of the organic hydroperoxide resistance protein Ohr [2]. OhrR is a transcriptional repressor classified in the MarR family. Under physiological conditions, the OhrR dimer binds the target promoter and represses transcription. When bacteria are exposed to organic hydroperoxides, OhrR is oxidized through the oxidation of a sensing reactive cysteine residue [7, 8]. Consequently, oxidized OhrR changes its structure and is released from the repressor binding site near the *ohr* promoter, thereby allowing the transcription of *ohr*. On the basis of cysteine residues involved in the oxidation step, bacterial OhrR proteins can be divided into two groups, 1-Cys and 2-Cys. The majority of the characterized OhrRs, including *Xanthomonas campestris* OhrR, belong to the 2-Cys group [7, 8]. Oxidation of *X*. *campestris* OhrR involves 2 cysteine residues. Upon exposure to organic hydroperoxides, a reactive sensing cysteine residue (Cys22) is oxidized, and reactive sulfenic acid intermediates react with a resolving cysteine residue (Cys127) forming an inter-subunit disulfide bond. Formation of the disulfide bond causes rotation of the winged helix-turn-helix motif leading to the repressor-DNA dissociation. *Bacillus subtilis* OhrR is a representative of 1-Cys group that contains a reactive cysteine residue corresponding to the Cys22 of the *X*. *campestris* OhrR [9]. Oxidation of the *B*. *subtilis* OhrR occurs through cysteine oxidation leading to formation of a mix-disulfide bond with a low-molecular-weight thiol molecule, bacillithiol [10, 11]. Experiments *in vivo* have demonstrated that OhrRs preferentially sense organic hydroperoxides ranging from synthetic alkyl hydroperoxide to fatty acid hydroperoxides. We have reported the characterization of *A*. *tumefaciens* OhrR, a member of the 2-Cys group OhrR that regulates a divergently transcribed gene *ohr* in an organic hydroperoxide-inducible fashion [2]. Currently, the only known OhrR target gene in *A*. *tumefaciens* is *ohr*.

In this communication, we functionally characterized *estR*, an *ohrR* paralog, as the transcriptional regulator of *estC*, a gene encoding a protein with esterase activity that belongs to the α/β hydrolase superfamily. The α/β hydrolases are one of the largest groups of structurally related enzymes containing an α/β hydrolase fold that consists of an eight-stranded, mostly parallel α/β structure and a Nucleophile-His-Acid catalytic triad [12]. The enzymes in this family catalyze diverse reactions and include acid ester hydrolase, haloperoxidase, haloalkane dehalogenase, and C-C bond breaking enzymes. We show here that *estC* expression is inducible by treatment with organic hydroperoxide.

## Results and Discussion

### The *atu5211* gene encodes an OhrR paralog

**Fig 1:**
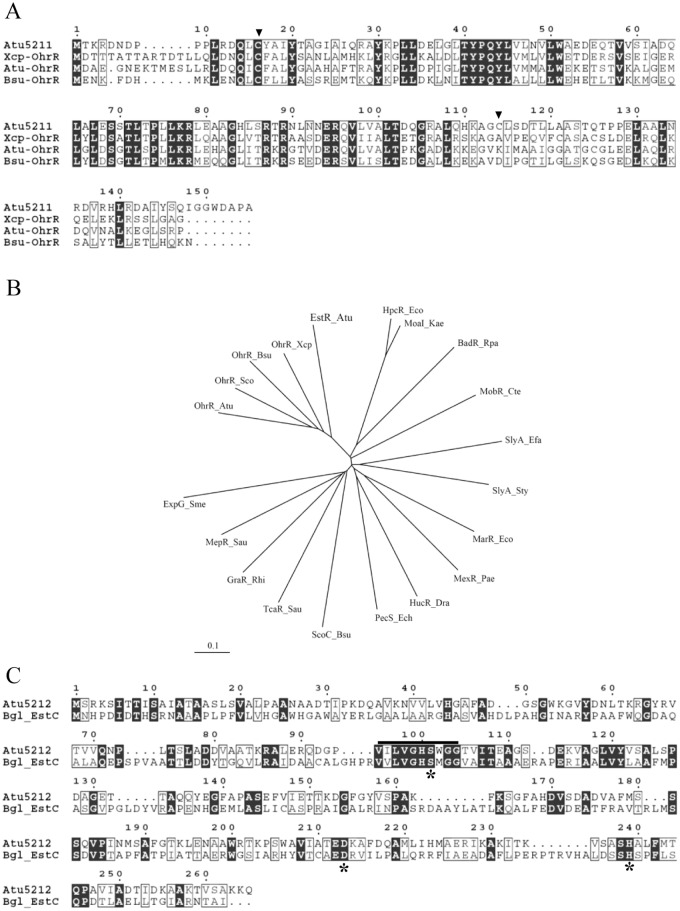
Multiple alignments of Atu5211 (EstR) and Atu5212 (EstC). A, The deduced amino acid sequence of Atu5211 (EstR) was aligned with OhrR from *Xanthomonas campestris* pv. phaseoli (OhrR_Xcp), *A*. *tumefaciens* (OhrR_Atu) and *Bacillus subtilis* (OhrR_Bsu). Cysteine residues are marked by arrow heads. B, A phylogenetic tree constructed from the amino acid sequences of transcriptional regulators belonging to MarR super family. EstR_Atu, *A*. *tumefaciens* EstR; OhrR_Xcp, *Xanthomonas campestris* pv. phaseoli OhrR (AAK62673); OhrR_Bsu, *Bacillus subtilis* OhrR (NP_389198); OhrR_Sco, *Streptomyces coelicolor* OhrR (CAB87337); OhrR_Atu, *Agrobacterium tumefaciens* OhrR (AAK86653); ExpG_Sme, *Sinorhizobium meliloti* ExpG (CAB01941); MepR_Sau, *Staphylococcus aureus* MepR (AAU95767), GraR_Rhi, *Rhizobium sp* GraR (BAF44528); TcaR_Sau, *Staphylococcus aureus* TcaR (AAG23887); ScoC_Bsu, *Bacillus subtilis* ScoC (NP_388880); PecS_Ech, *Erwinia chrysanthemi* PecS (CAA52427); HucR_Dra, *Deinococcus radiodurans* HucR (NP_294883); MexR_Pae, *Pseudomonas aeruginosa* MexR (AAO40258); MarR_Eco, *Escherichia coli* MarR (ABE11597); SlyA_Sty, *Salmonella typhimurium* SlyA (NP_460407); SlyA_Efa, *Enterococcus faecalis* SlyA (NP_816617); MobR_Cte, *Comamonas testosterone* MobR (BAF34929); BadR_Rpa, *Rhodopseudomonas palustris* BadR (NP_946008); MoaI_Kae, *Klebsiella aerogenes* MoaI (BAA09790); and HpcR_Eco, *Escherichia coli* HpcR (AAB25801). C, The deduced amino acid sequence of Atu5212 (EstC) was aligned with EstC from *Burkholderia gladioli* EstC [38]. The Ser-His-Asp catalytic triad residues are indicated by asterisks. The bar above the sequences represents the esterase-lipase active domain. Alignment was performed using ClustalW [32].

The *Xanthomonas campestris* OhrR sequence [13] was used to search the *A*. *tumefaciens* genome [14]. Five OhrR paralogs could be identified from 20 putative coding sequences (CDS) classified in MarR family. The results are atypical because most bacterial genomes have only one OhrR. We have previously characterized the OhrR (Atu0846) that regulates *ohr* (Atu0847) [2]. Here, an additional OhrR paralog, Atu5211 was identified. Multiple alignments of the Atu5211 sequence with other OhrRs revealed that key amino acid residues, particularly the peroxide sensing Cys16 residue that corresponds to Cys22 of *X*. *campestris* OhrR [13] and the Tyr30 and Tyr41 residues (Tyr36 and Tyr47 in *X*. *campestris* OhrR), which are important for sensing oxidized cysteine and forming a hydrogen bond network with Cys22 [8], are conserved (Fig 1A). The presence of an additional cysteine residue (Cys114) near the C-terminus suggests that Atu5211 belongs to the 2-Cys group of OhrRs [2, 13]. Phylogenetic analysis of selected regulators belonging to a MarR family strongly suggests that this Atu5211 is a transcription repressor (Fig 1B). However, Atu5211 is a distant member of other well characterized OhrRs (Fig 1B). This raises the question of whether mechanistically and functionally, Atu5211 is similar to other members of the OhrR group.

### Atu5211 is a transcriptional repressor of *atu5212*

One of the characteristics of the OhrR group of regulators is that they act as transcriptional repressors of the nearby target genes [2, 9, 13, 15, 16]. An analysis of the genes in proximity to *atu5211* revealed that it located and transcribed divergently from *atu5212*, which encodes a conserved hypothetical protein of 229 amino acids. In addition, *atu5211* is arranged head to tail with the *atu5210-5209* operon [14]. If Atu5211 regulates the expression of *atu5212* or *atu5210-5209* operon, we would expect that inactivation of *atu5211* should lead to an increase in its basal expression level. Thus, the expression levels of *atu5212* and *atu5210-5209* were determined in an *atu5211* mutant and in its parent, NTL4 (data not shown). Only the *atu5212* basal expression levels were increased more than 10-fold in the *atu5211* mutant. The expression of *atu5210* did not significantly increase in the mutant compared to NTL4. This indicates that Atu5211 acts as a transcription repressor of *atu5212* expression.

### *atu5210*, *atu5211* and *atu5212* are designated *scd*, *estR* and *estC*

A KEGG SSDB search [17] for orthologous proteins of Atu5212 in closely related bacteria revealed that this putative protein was an N-terminally truncated protein compared to other orthologous proteins that share high scores of identity. Analysis of nucleotide sequences upstream of the putative translation initiation (GTG) suggested a new potential ATG codon located 105 nucleotides from the original annotated GTG. This places the start codon in a good position from the transcription start site of *atu5212* determined in the *estR*-*estC* promoter characterization section. The re-annotated Atu5212 ORF encodes a 265-amino-acid protein. The NCBI conserved domain search [18] of the deduced amino acid sequence of Atu5212 identified two domains of the esterase-lipase superfamily and the C-C hydrolase MhpC. Scanning for the active domain using InterProScan [19] demonstrated a lipase serine active site domain (IPR008262) located at amino acid residues 95–104 (VILVGHSWGG), which consists of a catalytic triad of nucleophile-His-acid amino acid [12] as Ser101-Asp213-His239 (Fig 1C). The nucleophilic serine is situated in a highly conserved GXSXG pentapeptide motif that corresponds to the sequence motif GHSWG in Atu5212. In addition, the conserved CGHWA/T motif of the MhpC family was absent from the Atu5212 sequence [20]. These features, together with the amino acid sequence surrounding the active His239, ASH_239_AL, suggests that Atu5212 belongs to an esterase-lipase family rather than an MhpC family. Henceforth, the Atu5212 is designated as EstC. The data from the expression pattern of *estC* indicated that Atu5211 acts as a transcriptional repressor of *estC*; hence, *atu5211* is designated *estR* for "regulator of an esterase gene". The BLAST search of the Atu5210 sequence indicates it has homology to a family of short chain dehydrogenases and thus is designated Scd.

### Genome organization of *scd-estR-estC*

**Fig 2:**
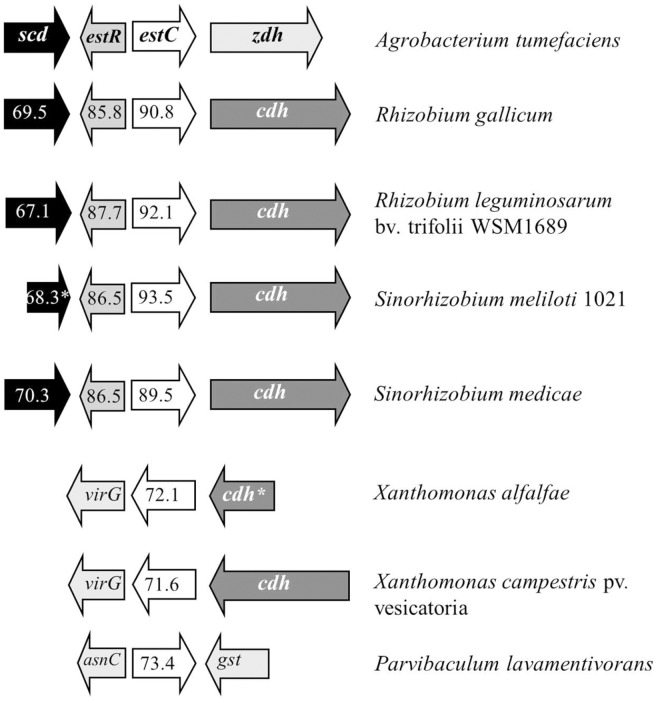
Gene organization of the *estC* locus in various bacteria. The arrow indicates the orientation of the transcription. The number in the arrows represents percentage of identity of each sequence to the corresponding *A*. *tumefaciens* genes. The asterisk indicates a truncated gene. *scd*, short chain dehydrogenase; *zdh*, zinc dehydrogenase; *cdh*, choline dehydrogenase; and *gst*, glutathione S-transferase.

Analysis of the genome organization of these genes in different bacteria revealed a conserved organization that showed *estC* homologous genes located next to the ORFs that shared roughly 90% identity to *estR* (Fig 2). The gene arrangement consisting of *scd* (short chain dehydrogenase)-*estR*-*estC*-*zdh* or *cdh* (zinc or choline dehydrogenase) is well conserved in *Rhizobium* and *Agrobacterium* bacteria. The genes within this arrangement also shared a high percent identity within the bacterial groups. This conserved gene organization in these bacteria raised the possibility that it could arise from horizontal gene transfer among alphaproteobacteria living in soil.

Nevertheless, ORFs sharing greater than 70% amino acid sequence identity to *A*. *tumefaciens* EstC are found in non-alphaproteobacteria, including *Xanthomonas campestris* pv. vesicatoria (XCV2148), *Xanthomonas alfalfa* and *Parvibaculum lavamentivorans* (Fig 2). In these phylogenetically distant bacteria, the *estC* homologs are not always located next to EstR homologs (Fig 2). In *Xanthomonas* spp., *estC* is organized in head-to-tail fashion with the *cdh* genes.

### *estC* encodes an esterase

Analysis of the EstC sequence suggests that it encodes an esterase/lipase enzyme. The observation was extended by investigating the enzymatic activity of EstC. The C-terminal 6×His-tagged EstC fusion protein was purified from *E*. *coli* using Ni-NTA affinity column chromatography. The purity of EstC was greater than 95% as estimated by densitometric scanning of the Coomassie blue stained protein in polyacrylamide gels (as shown in S1 Fig). The esterase activity of purified EstC was measured using several *p*-nitrophenyl ester substrates including *p*-nitrophenyl butyrate (C4), *p*-nitrophenyl decanoate (C10), and *p*-nitrophenyl palmitate (C16) in assay reactions performed as previously described [21] and in the Methods section. EstC had minimal activity when *p*-nitrophenyl decanoate (C10, 3,446 ± 301 U mg^-1^ protein) and *p*-nitrophenyl palmitate (C16, 295 ± 86 U mg^-1^ protein) were used as substrates. The results suggest, based on the EstC enzymatic efficiency towards different chain length substrates, that EstC is an esterase rather than a lipase.

**Fig 3:**
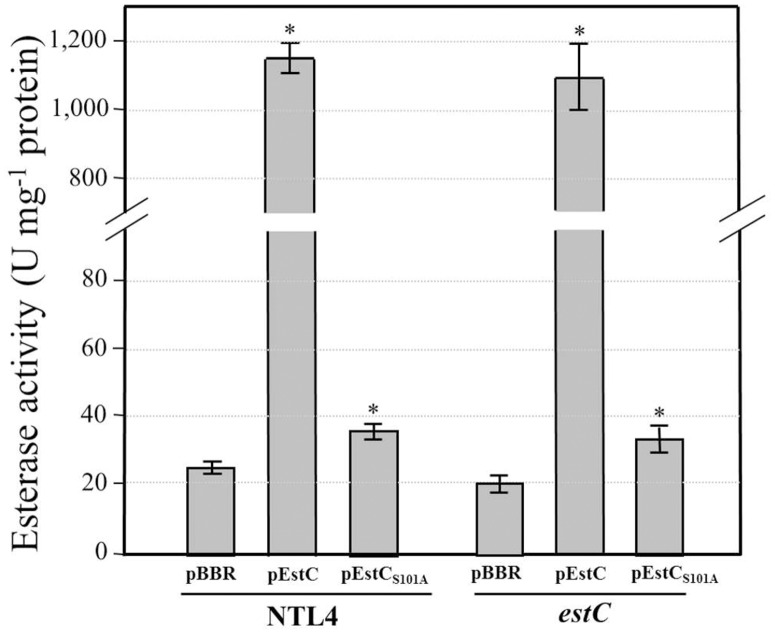
Esterase activity in *A*. *tumefaciens* NTL4 and derivatives. The esterase activity in NTL4 and the *estC* mutant strains harboring the pBBR1MCS-5 vector control (pBBR), pEstC or pEstC_S101A_ was assayed in crude lysates prepared from exponential phase cultures. A unit of esterase activity is defined as the amount of enzyme capable of hydrolyzing *p*-nitrophenyl butyrate to generate 1 μmol of *p*-nitrophenol at 25°C. Asterisks indicate a significant difference (*P* < 0.05) from NTL4 or the *estC* mutant harboring vector control.

Esterase activity using *p*-nitrophenyl butyrate (C4) as the substrate was monitored in various *A*. *tumefaciens* strains. EstC mutant and *estC* high expression strains were constructed. The *estC* mutant strain showed 20% less esterase activity (19.8 ± 3.0 U mg^-1^ protein) than NTL4 (25.4± 1.9 U mg^-1^ protein). The complemented strain (*estC*/pEstC) produced 1,101 ± 94 U mg^-1^ protein, similar to the level, 1,149 ± 47 U mg^-1^ protein, attained in NTL4 harboring ectopic *estC* (NTL4/pEstC) (Fig 3). The finding that esterase activity was retained at relatively high levels in the *estC* mutant strongly suggested that *A*. *tumefaciens* produces other proteins with esterase activity. Analysis of the *A*. *tumefaciens* genome revealed the presence of an ORF (Atu5066) that shares 35% identity to EstC. Atu5066 is located on the pAT megaplasmid. Its function and gene regulation is being investigated. Analysis of the EstC primary amino acid sequence revealed a putative catalytic triad composed of Ser101-Asp213-His239. Ester hydrolysis is initiated by a nucleophilic attack of the catalytic site at a Ser residue, and the importance of Ser101 to the esterase activity was investigated by a site directed mutagenesis of *estC* that changed the active site Ser101 to Ala (S101A). The mutated *estC*_S101A_ was cloned into an expression vector to generate pEstC_S101A_. This recombinant plasmid was transferred into NTL4, and the resulting esterase activity was determined. The NTL4 harboring pEstC_S101A_ produced esterase activity of 35.6 ± 2.7 U mg^-1^ protein, whereas expression of wild-type *estC* expressed from the same plasmid vector (pEstC) generated 1,149 ± 47 U mg^-1^ protein esterase activity (Fig 3). This suggests that Ser101 plays a crucial role in the esterase activity of EstC.

### The phenotypes of *estR* and *estC* mutants

**Fig 4:**
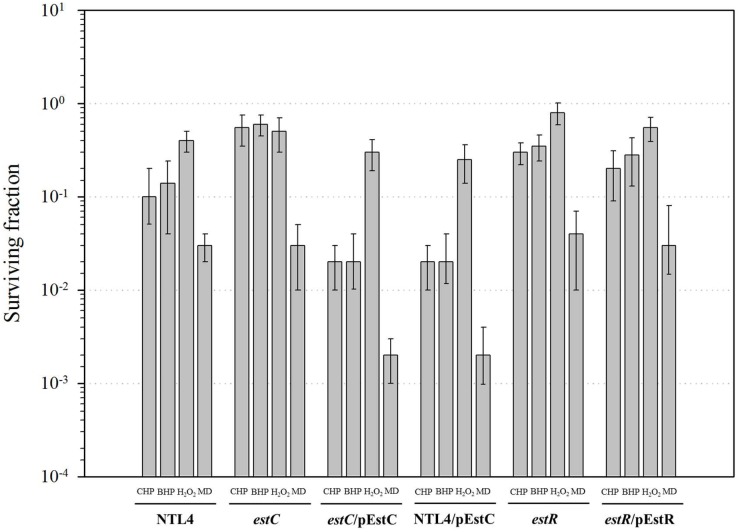
Phenotypic analysis of *A*. *tumefaciens* NTL4 and derivatives. The oxidant resistance levels of *A*. *tumefaciens* NTL4, the *estC* mutant, the complemented *estC* mutant (*estC*/pEstC), NTL4 harboring pEstC (NTL4/pEstC), the *estR* mutant, and the complemented *estR* mutant (*estR*/pEstR) were determined using plate sensitivity assays as described in the Methods. The concentrations of oxidant used are 0.25 mM CHP, 1.2 mM BHP, 0.35 mM H_2_O_2_, and 0.55 mM MD. The survival colonies were counted after 24 h incubation at 30°C. The surviving fraction is defined as the number of colony forming units (CFU) on plates containing oxidant divided by the number of CFU on plates without oxidant.

The finding that *estC* was regulated by *estR*, a putative organic hydroperoxide-inducible repressor, suggested that this gene system could have physiological roles in oxidative stress resistance. The resistance levels of *A*. *tumefaciens* NTL4 and the *estC* and *estR* mutants to oxidants were determined using plate sensitivity assays. The results showed that the *estC* mutant was 5-fold more resistant than NTL4 to cumene hydroperoxide (CHP) and *t*-butyl hydroperoxide (BHP), whereas the resistance levels toward H_2_O_2_ and superoxide generator menadione (MD) in NTL4 and the *estC* mutant were not significantly different (Fig 4). Moreover, we observed that in strains NTL4/pEstC and *estC*/pEstC, which highly expressed *estC*, were 10-fold less resistant to CHP, BHP and MD. An *estR* mutant had a small (less than 3-fold) decrease in the resistance levels to CHP, BHP and MD (Fig 4). The observations suggest that there are correlations between the expression levels of *estC* and the bacterial resistance levels to organic hydroperoxides.

### EstR regulates organic hydroperoxide-inducible *estC* and *estR* expression

**Fig 5:**
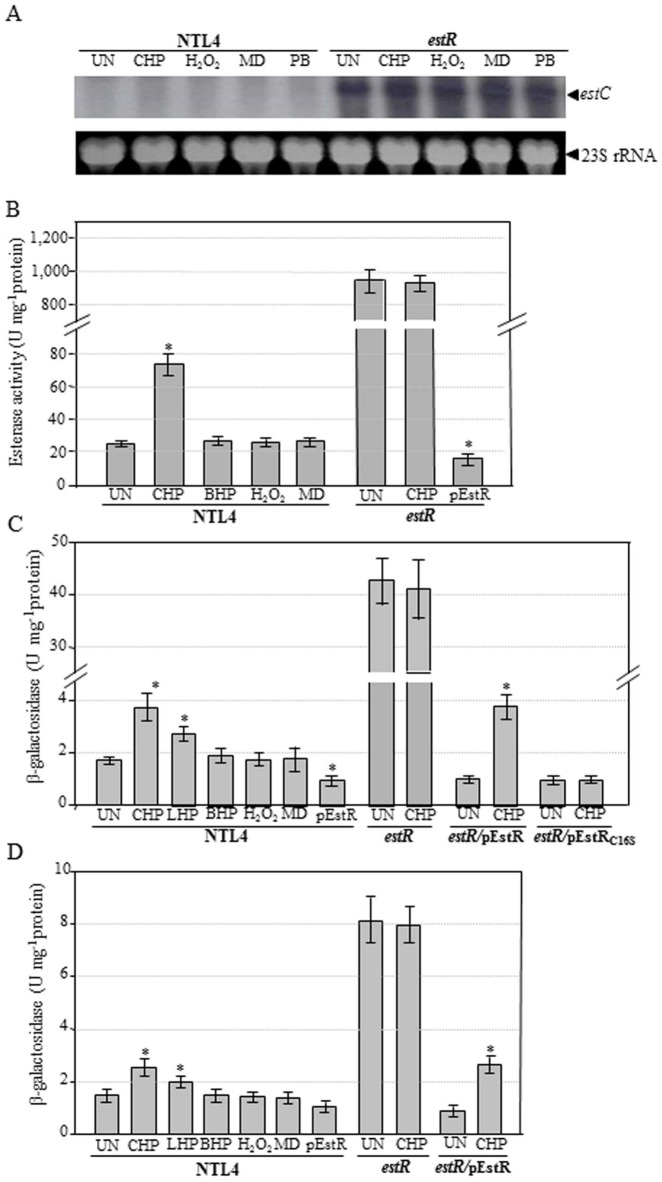
Expression analysis of *estC*. A, Northern analysis of *estC* in *A*. *tumefaciens* NTL4 wild-type and the *estR* mutant was performed using the [^32^P]-labeled *estC* probe. Equal amounts of RNA (10 μg) prepared from bacterial cultures grown under uninduced (UN) conditions or induced with 1 mM CHP, 250 μM H_2_O_2_, 200 μM MD or 250 μM plumbagin (PB) were used in the experiments. The 23S rRNA used as the amount control is shown below the hybridized autoradiograph. B, Esterase activity in *A*. *tumefaciens* NTL4 and the *estR* mutant strains was assayed in crude lysates prepared from cultures cultivated as described in A. BHP, culture induced with 500 μM *t*-butyl hydroperoxide. C and D show the *estC* and *estR* promoter activities *in vivo*, respectively, in NTL4, the *estR* mutant, and the complemented strain (*estR*/pEstR) carrying the plasmid either pP_estC_ that contains the *estC* promoter-*lacZ* fusion (C) or pP_estR_ that contains the *estR* promoter-*lacZ* fusion (D). The activities were monitored in samples of lysates prepared from uninduced (UN) and oxidant-induced cultures (1 mM CHP, 25 μM LHP, 500 μM BHP, 250 μM H_2_O_2_, and 250 μM MD). The β-galactosidase activity is expressed in international units. pEstR means harboring the *estR* expression plasmid. Asterisks indicate a significant difference (*P* < 0.05) from the uninduced control.

Transcriptional regulators of OhrR subgroup are involved in the regulation of organic hydroperoxide-inducible genes [22]. Previous findings in other bacteria indicate that OhrR homologs often regulate expression of genes in their close proximity. The Northern analysis of *estC* in RNA samples extracted from the NTL4 wild-type and an *estR* mutant cultivated under uninduced conditions and induced with oxidants was performed. In NTL4, the level of *estC* transcripts in the uninduced sample was too low to be detected by the Northern blot (Fig 5A). A barely detectable band of *estC* in the CHP induced RNA sample was observed, but not from other conditions (Fig 5A and S2 Fig). It was established that EstR was a repressor of *estC*. This notion was confirmed by Northern analysis of *estC* expression performed on RNA samples from the uninduced and oxidant induced cultures of the *estR* mutant. The results showed that in the mutant, *estC* expression was high in uninduced and in oxidant induced samples (Fig 5A).

Because *estC* encodes an esterase, the total esterase activity of NTL4 uninduced and oxidant induced cultures was assayed. As expected, CHP treatment induced a 3-fold increase in the total esterase activity (73.0 ± 6.8 U mg^-1^ protein) compared with the uninduced level (25.4 ± 1.9 U mg^-1^ protein) in NTL4 (Fig 5B). Treatment of cultures with other oxidants, including BHP, H_2_O_2_ and MD, did not induce the esterase activity. The total esterase activity was determined in cultures of the *estR* mutant. The results showed that the *estR* mutant had 37-fold more total esterase activity compared to NTL4 (Fig 5B). No significant induction of esterase activity by CHP treatment of the mutant was observed.

Next, *estC* promoter activity was measured *in vivo* using the *estC* promoter-*lacZ* fusion. The plasmid pP_estC_ carrying the *estC* promoter fragment fused to *lacZ* was introduced into NTL4 and the *estR* mutant. The level of β-galactosidase activity was monitored in uninduced and CHP induced NTL4/pP_estC_ (Fig 5C). Significant induction of β-galactosidase activity was detected in NTL4/pP_estC_ treated with CHP (3.7 ± 0.4 U mg^-1^ protein) compared with the uninduced sample (1.8 ± 0.3 U mg^-1^ protein) (Fig 5C). CHP-induced *estC* expression was abolished in the *estR* mutant. The level of β-galactosidase activity in *estR*/pP_estC_ was constitutively high (Fig 5C). In the *estR* complemented strain (*estR*/pP_estR_), the *estC* promoter activity (1.0 ± 0.1 U mg^-1^ protein) dropped to a level below the NTL4 level (1.8 ± 0.3 U mg^-1^ protein) and 40-fold below the level attained in the *estR* mutant strain (43.0 ± 4.4 U mg^-1^ protein) (Fig 5C). CHP treatment was able to induce the expression of the *estC* promoter in *estR* complemented strain (Fig 5C). Taken together, the results support the role of EstR as an organic hydroperoxide sensor and repressor of *estC*.

Nonetheless, the inability of BHP treatment to induce *estC* expression even at a high concentration (1 mM) was unexpected. Together with the fact that the maximal level of *estC* promoter activity in NTL4 induced with CHP (3.7 ± 0.4 U mg^-1^ protein) was much lower than in the *estR* mutant (43.3 ± 4.4 U mg^-1^ protein), the question arose whether organic hydroperoxide is the preferred inducer of *estC*. A number of substances were tested for their ability to induce *estC* expression using pP_estC_, a *lacZ* fusion construct. The results indicated that cumyl alcohol (a compound structurally related to CHP and a metabolite of CHP metabolism), salicylic acid (a strong inducer of the MarR transcription regulator), perbenzoic acid, methyl jasmonate (an ester substance that plants produce as a signal molecule during plant-microbe interaction), the esterase substrates (*p*-nitrophenyl butyrate, *p*-nitrophenyl decanoate, and *p*-nitrophenyl palmitate), and linoleic acid failed to induce *estC* expression (data not shown). Moreover, oxidants, including H_2_O_2_ and MD, failed to induce the expression of *lacZ* from pP_estC_ in NTL4 (Fig 5C). Treatment of NTL4/pP_estC_ with 25 μM linoleic hydroperoxide (LHP) induced moderate levels of *estC* expression (2.7 ± 0.3 U mg^-1^ protein), while no induction was observed in BHP-treated cells (Fig 5C). The results suggest that EstR could be more readily oxidized by the hydrophobic organic hydroperoxides (CHP and LHP) leading to its structural alteration and subsequent inability to bind to operator sites. This allows inducible *estC* expression. A less hydrophobic organic hydroperoxide such as BHP could not efficiently oxidize the regulator and hence would be unable to induce the expression of the gene.

The expression of genes in the *marR* family is typically autoregulated [13, 23, 24]. To test whether *estR* regulates its own expression *in vivo*, a promoter analysis using a promoter-*lacZ* gene fusion was conducted. A pP_estR_ plasmid that contains a putative *estR* promoter sequence transcriptionally fused to a promoterless *lacZ* was introduced into *A*. *tumefaciens* NTL4 and the *estR* mutant. The levels of β-galactosidase activity in lysates prepared from cultures of NTL4 and the *estR* mutant harboring pP_estR_ grown under uninduced and oxidant induced conditions were monitored. The *estR* promoter activity in NTL4 was induced only in cells treated with CHP (Fig 5D). CHP induced a 1.6-fold increase in β-galactosidase activity relative to the uninduced sample. The *estR* promoter was constitutively active in the *estR* mutant compared to that in the wild-type (Fig 5D). The promoter activity of *estR* in the complemented strain (*estR*/pEstR) showed that pEstR could repress the constitutively high activity of the *estR* promoter in the *estR* mutant to the level attained in the NTL4 strain (Fig 5D). It is noteworthy that a high expression level of *estR* in the NTL4/pEstR lowered the level of *estR* promoter activity (Fig 5D). This is consistent with the notion that higher levels of the repressor lead to greater repression of target gene expression. The evidence supports the role of EstR as an autoregulated, organic hydroperoxide-inducible, transcriptional repressor.

We extended the investigation to determine whether EstR would cross regulate *ohr* expression using an *estR* mutant. The transcription activity of pP_ohr_ [2] that had the *ohr* promoter transcriptionally fused to a *lacZ* reporter gene in the mutant (*estR*/pP_ohr_) and in NTL4 (NTL4/pP_ohr_) was determined under uninduced and organic hydroperoxide induced conditions by monitoring β-galactosidase activity. As expected, β-galactosidase activities showed CHP induction in both *estR* and NTL4 suggesting that *estR* plays no role in *ohr* expression (data not shown). There is no cross-regulation between the two members of the MarR family.

### EstR senses organic hydroperoxides through Cys16

EstR possesses a conserved sensing cysteine residue at position 16 (Cys16). We tested whether Cys16 of the EstR is involved in organic hydroperoxide sensing mechanism by constructing a site specific mutation changing Cys16 to serine (C16S). The plasmid pEstR_C16S_ was introduced into the *estR* mutant harboring pP_estC_. As shown in Fig 5C, the basal level of *estC* promoter activity in *estR*/pEstR_C16S_/pP_estC_ was similar to the level attained in the *estR*/pEstR/pP_estC_ strain. Thus, the mutation C16S did not affect the ability of EstR_C16S_ to bind to operator regions and repress *estC* gene expression. Nevertheless, no CHP-induced *estC* promoter activity was observed in the *estR* mutant expressing *estR*_C16S_, indicating that Cys16 of EstR has a crucial role in the organic hydroperoxide sensing process. The mutant complemented with wild type *estR* (*estR*/pP_estC_/pEstR) showed CHP induction of the promoter (Fig 5C). Other transcriptional regulators in the OhrR group, including *Pseudomonas aeruginosa* OspR [22] and *Staphylococcus aureus* AbfR [25], also show peroxide or oxidant sensing processes that occur through the initial oxidation of the sensing cysteine residue located near the N-terminus [26]. In the 2-Cys group of OhrR, after the initial oxidation of the sensing Cys by organic hydroperoxide, there are important amino acids residues, including Tyr36 and Tyr47, involved in sensing oxidized Cys and maintaining the hydrogen bond environment around the sensing Cys. These amino acids are important for the subsequent formation of a disulfide bond with a resolving Cys near the carboxyl terminus and for the accompanying structural changes. These conserved residues are also found in EstR.

The data clearly show that the oxidation of the sensing residues of EstR is required to initiate structural modifications that prevent the repressor from binding to the operator sites. Hence, molecules that do not have a hydroperoxide moiety could not act as inducers of the EstR system. Interestingly, the pattern of induction of *A*. *tumefaciens* OhrR and EstR are clearly different. OhrR can be oxidized by simple BHP [2], whereas EstR can only be oxidized by higher concentrations and by more hydrophobic hydroperoxides, including CHP and LHP. Analysis of the structure of a 2-Cys member of OhrR reveals the sensing Cys residue (corresponding to Cys16 in EstR) buried down in a hydrophobic pocket [8]. This is thought to favor the hydrophobic organic hydroperoxide to move down the pocket and oxidize the sensing Cys residue, which accounts for the observation that organic hydroperoxides are more efficient inducers than H_2_O_2_. It is possible that in EstR the sensing Cys (C16) is less accessible and coupled with a regulator that might have a higher oxidation potential than *A*. *tumefaciens* OhrR so that it could be oxidized only by either relatively higher concentrations or more hydrophobic organic hydroperoxides.

### Characterization of the *estR-estC* promoters

**Fig 6:**
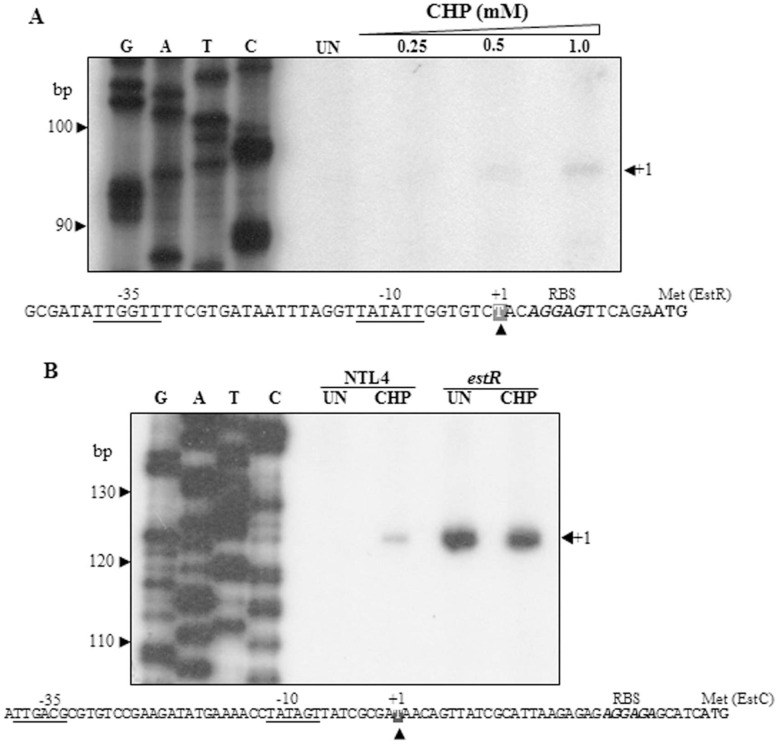
Characterization of the *estR* and *estC* promoters. Primer extension experiments with *estR* (A) and *estC* (B) were conducted using mRNA from *A*. *tumefaciens* NTL4 and the *estR* mutant. The experiment was performed using the [^32^P]-labeled BT1575 primer for *estR* and the BT1574 primer for *estC* and 5 μg total RNA extracted from uninduced (UN) or CHP-induced cultures. G, A, T, C are sequence ladders of pGEM-3Zf (+) prepared using the pUC/M13 forward primer, and numbers represent the size in base pairs of the sequencing products. +1 indicates the transcription start site. The -10 and -35 regions are underlined. RBS and Met represent the ribosome binding site and the translation start site, respectively.

The 5’ end of the *estR* transcript was determined using primer extension with the [^32^P]-labeled BT1575 primer and total RNA extracted from NTL4 wild-type uninduced cultures and cultures induced with various concentrations of CHP. The extension products were separated by denaturing PAGE. As shown in Fig 6A, a principal primer extension product of 95 nucleotides was detected in RNA samples from the cultures induced with 0.5 and 1 mM CHP. No primer extension products could be detected in the RNA from uninduced cultures or cultures induced with 0.25 mM CHP. The size of the RNA product corresponded to transcription initiation at the T residue located 14 bases upstream of the ATG codon. The *E*. *coli* RNA polymerase σ ^70^-like -35 and -10 sequence elements, TTGGTT and TATATT, separated by 17 bases could be found upstream of the +1 site. The primer extension results are in good agreement with the results of the *estR*-*lacZ* promoter fusion (Fig 5D). Interestingly, the concentrations of CHP required to induce *estR* expression were relatively high (1 mM) compared with the concentration (50 μM) required for the induction of *ohrR* expression [2].

To characterize the *estC* regulatory elements, the transcription start site of *estC* was mapped using primer extension experiments performed with [^32^P]-labeled BT1574 and RNA samples prepared from the cultures of either NTL4 or the *estR* mutant. The extension product of 123 nucleotides corresponded to the putative +1 of *estC* located at T, 34 nucleotides upstream of the ATG translational start codon of *estC*. The putative -10 and -35 elements of the *estC* promoter were mapped as TATAGT and TTGACG, respectively, separated atypically by 20 bp (Fig 6B). The primer extension results showed that the RNA from CHP induced cultures produced much higher level primer extension products than the samples from uninduced cultures. This supported the results of the Northern blot analysis, the *estC* promoter fusion analysis and the total esterase assays showing that *estC* expression was inducible by CHP treatment. Experiments showed that the *estR* mutant exhibited constitutively high levels of *estC* primer extension products in RNA samples from both uninduced and CHP-induced cultures (Fig 6B). The levels were more than 30-fold higher than the level attained in NTL4, confirming the results of the Northern analysis of *estC* expression, the *estC* promoter fusion and the esterase activities in the *estR* mutant.

### Binding of purified EstR to the *estR* and *estC* promoters

**Fig 7:**
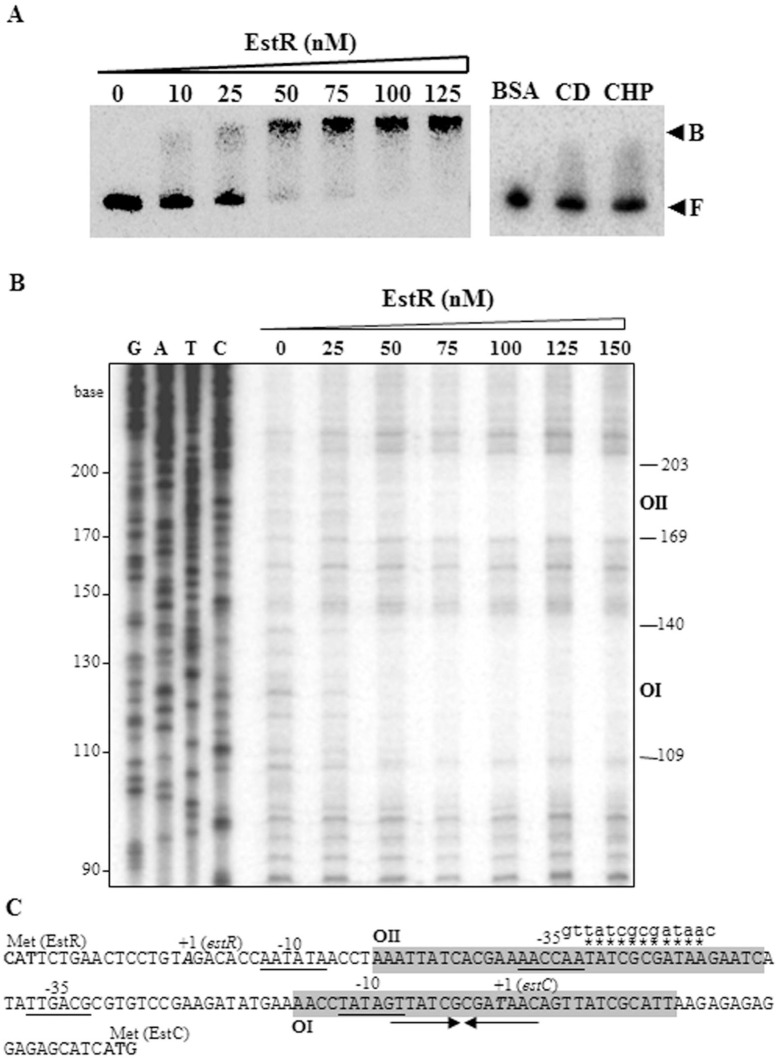
Gel mobility shift assays and DNase I footprinting of EstR bound to the *estR-estC* promoters. A, Gel mobility shift assays of the [^32^P]-labeled 314-bp DNA fragment spanning the *estR* and *estC* promoters with purified EstR protein (0–125 nM) were performed. The complexes were separated on native PAGE. The specificity of the binding was validated by adding the cold DNA fragment (CD) to the binding mixture or adding bovine serum albumin (BSA) to the reaction instead of the EstR protein. CHP represents the bound complexes treated with 1 mM CHP. B and F indicate bound and free probes, respectively. B, DNase I protection assays were performed with the [^32^P]- labeled *estR-estC* promoter fragment and purified EstR protein at the indicated concentrations. The digested and protected DNA fragments were separated on 8% denaturing DNA sequencing gels beside the sequence ladders (G, A, T, C) generated from pGEM-3Zf (+) using the pUC/M13 forward primer. The numbers on the left side are the size in base pairs of the bands. C, The depicted sequence shows the *estR-estC* promoter region. +1 and Met indicate the transcription and translation start sites. The -10 and -35 regions are underlined. RBS represents the ribosome binding site. The sequences corresponding to the sites of OI and OII protection are shaded. Arrows indicate palindromic sequences. Small letters above the sequence line represent the putative EstR binding box derived from site OI protection. Identical nucleotides are marked by asterisks.

The expression profiles of *estR* and *estC* strongly suggest that these two promoters are under the control of EstR. To assess the ability of purified EstR protein to bind to the *estR* and *estC* promoter regions, DNA mobility shift assays were conducted. EstR protein was purified using a heparin column, and its purity was assessed to be greater than 90% (S3 Fig). With regard to the *estC* and *estR* promoter characterization, the -35 elements of *estR* and *estC* are separated by 19 bases, hence the 314-bp DNA sequence encompassing both promoters was used in the experiment. Purified EstR protein (10–125 nM) was incubated with the [^32^P]-labeled promoter fragment in a binding buffer. The protein-DNA complex was observed at an EstR concentration of 25 nM and was maximal at 100 nM (Fig 7A). The binding affinity of purified EstR is similar to previously characterized OhrR members binding to their operators [2, 27, 28]. The specificity of the EstR binding was evaluated, and the results illustrated that cold promoter fragments competed with the labeled fragment for binding to the EstR protein, whereas an unrelated protein (BSA) was unable to bind the promoter fragment (Fig 7A). The results indicate that purified EstR protein specifically binds to the *estR-estC* promoter fragment *in vitro*. Binding of EstR therefore represses the expression of *estC* as well as its own expression. To ascertain whether CHP treatment is able to release the EstR from the promoter, the complex was exposed to 1 mM CHP. As shown in Fig 7A, prior CHP treatment of EstR prevented the repressor from binding to the promoter fragment as shown by the absence of the complex. Thus, induction of *estC* and *estR* by CHP is a consequence of the oxidation and subsequent structural changes of EstR that prevent the repressor from binding to the promoters, thereby allowing transcription of *estC* and *estR*. The observation fits well with the model of OhrR transcriptional repressors that the reduced repressor binds to the operator and inhibits transcription of the target promoter. The precise location of the EstR operator sites within the *estR-estC* intergenic region was determined by DNaseI footprinting using purified EstR and the [^32^P]-314-bp *estR-estC* promoter fragment. Binding reactions containing various concentrations of purified EstR were digested with DNaseI. The results demonstrated that EstR bound to two sites in the intergenic region of the *estR-estC* promoter in a region of +16 to -18 (site OI) and -46 to -82 (site OII) of the *estC* promoter (Fig 7B). The EstR operator binding site OI covered the -10 motif of the *estC* promoter. The binding of EstR to the OI site would block the binding of RNA polymerase to the *estC* promoter (Fig 7C). The EstR operator site OII overlaps with the -35 region of the *estR* promoter (Fig 7C). Hence, binding of EstR to OII blocks the -35 region of the promoter and prevents RNA polymerase from binding to the promoter. The binding affinities of EstR to OI and OII were slightly different. The DNaseI protection was observed at EstR concentrations of 50 nM and 75 nM for binding at OI and OII, respectively. This suggests that EstR binds to OI at a higher affinity than OII. Analysis of OI and OII revealed the presence of a 14-base-pair palindromic sequence, GTTATCGCGATAAC, and a homologous sequence, AATATCGCGATAAG, respectively (Fig 7C). The palindromic EstR operators OI and OII are quite different from the OhrR OI binding site (TACAATT-AATTGTA) identified in *A*. *tumefaciens* [2]. This is likely to be responsible for the observed lack of cross regulation between OhrR and EstR (data not shown). The EstR dual operator sites model here is distinct from the previously characterized *A*. *tumefaciens* OhrR regulation of the *ohrR-ohr* promoter region where only a single OhrR operator site regulates transcription of both *ohr* and *ohrR* [2].

Taken together, the results suggest that under physiological and uninduced conditions, EstR binds to the operator sites, resulting in steric hindrance of RNA polymerase binding to both the *estR* and *estC* promoters and thereby repressing their transcription. Upon exposure to organic hydroperoxide, oxidation of EstR decreases the concentration of reduced EstR and the binding of reduced EstR to the *estC* operator site OI and the *estR* operator site OII. This allows expression of *estC* and *estR*. As the concentration of inducer declines and the concentration of reduced EstR concomitantly increases, the repressor separately binds to both operators, repressing the expression of both genes and returning the expression of both genes to the uninduced state.

## Methods

### Bacterial growth conditions

*Agrobacterium tumefaciens* NTL4, a pTiC58-cured derivative of C58, Δ*tet*_C58_ [29], and its mutant and complemented derivatives were cultivated in Lysogeny Broth (LB) medium and incubated aerobically at 28°C with continuous shaking at 150 rpm. The overnight cultures were inoculated into fresh LB medium to give an optical density at 600 nm (OD_600_) of 0.1. Exponential phase cells (OD_600_ of 0.6 after 4 h of growth) were used in all experiments. The oxidant-induced experiments were performed by challenging the exponential phase cultures with 250 μM to 1.0 mM cumene hydroperoxide (CHP), 250 μM H_2_O_2_, 500 μM *tert*-butyl hydroperoxide (BHP) or 200 μM menadione (MD) for 15 min for Northern analysis and 30 min for enzymatic assays.

### Molecular biology techniques

General molecular genetics techniques, including genomic and plasmid DNA preparation, RNA preparation, Southern and Northern blot analyses, PCR, cloning, transformation into *Escherichia coli*, and gel electrophoresis, were performed using standard protocols [30]. *A*. *tumefaciens* was transformed by electroporation [29].

### Alignments and phylogenetic analyses

Amino acid sequences were retrieved from the GenBank database [31]. The alignments were performed by using the multiple alignment feature of ClustalW version 2.0.12 [32] with maximal fixed-gap and gap extension penalties and displayed using ESPript 3.0 (http://espript.ibcp.fr/ESPript/cgi-bin/ESPript.cgi). A phylogenetic tree was constructed by the neighbor-joining method based on ClustalW analysis data and displayed using PHYLODENDRON, version 0.8d (D.G. Gilbert, Department of Biology, University of Indiana, USA at http://iubio.bio.indiana.edu).

### Primer extension

Total RNA was extracted from wild-type *A*. *tumefaciens* and from the *estR* mutant cultivated under uninduced and cumene hydroperoxide-induced conditions. Primer extension experiments were performed using the primers [^32^P]-labeled BT1574 (for *estC*) or BT1575 (for *estR*) (see Table 1), 5 μg total RNA, and 200 U superscript III MMLV reverse transcriptase (Promega, USA). Extension products were analyzed on 8% acrylamide-7 M urea gels with sequencing ladders generated using a PCR sequencing kit with labeled M13F primer and pGem3Zf(+) plasmid as the template.

**Table 1:** List of primers

Primer	Sequence (5’→3’)
BT493	CAGGAGTTCAGAATGACCA
BT494	TACTCTTGCAGGTCAGGC
BT1505	ATCGCTGAGACAGCCTGC
BT1506	GGCGGAAGACGAGCAGAC
BT1507	CTCCGGACGCCGGCGAGAC
BT1508	CTTGGTGCCGAACGCAGAC
BT1559	CCGATGGCGCGACGTCGATCATTGC
BT1574	GTGTCGGCTGCATTCGCTGC
BT1575	CTGGATAGCGATGCCTGCGG
BT1595	CGCGTGTCCGAAGATATGAAAACCTA
BT1621	TCAGGTACCCCAAACGCGAT
BT1622	CTCTTCGAAGTCAGGCGGG
BT1632	CACCATGGCACGCAAGTCCAT
BT1633	GTGAGCTCTTGCTTTTTTGCTG
BT1662	GCCCCAGGCATGTCCGACGAG
BT1663	GTCGGACATGCCTGGGGCGGA
BT3859	CTGCGCGACCAGCTCAGCTATGCT
BT3860	TATAAATAGCATAGCTGAGCTGGT
M13F	GTAAAAGGACGCCCAGT

### Construction of *A*. *tumefaciens estR* and *estC* mutants

The *A*. *tumefaciens estR* mutant was constructed by insertional inactivation using the pKnock suicide vector [33]. An internal fragment of the *estR* gene was PCR amplified with the primers BT1505 and BT1506 (see Table 1) and *A*. *tumefaciens* genomic DNA as the template. The 199-bp PCR product was cloned into pDrive (Qiagen, France), and the nucleotide sequence of the insert was determined to assure it was indeed an *estR* fragment. Then, a *Bam*HI-*Hinc*II fragment was subcloned into pKnock-Gm digested with the same enzymes to form pKnock_estR_. This recombinant plasmid was transferred into *A*. *tumefaciens* and selected for the gentamicin resistance (Gm^r^) phenotype. The *estR* mutant was confirmed by Southern blot analysis.

The *estC* mutant was constructed using the same protocol described for the *estR* mutant. The *estC* fragment was amplified with the BT1507 and BT1508 primers (see Table 1) and cloned into pDrive. An *Eco*RI fragment was then subcloned into pKnock-Km [33] at the *Eco*RI site. The *estC* mutant was selected for kanamycin resistance (Km^r^) and verified by Southern blot analysis.

### Construction of the pEstR, pEstR_C16S_, pEstC and pEstC_S101A_ expression plasmids

To construct the pEstR expression plasmid, the full-length gene was amplified from *A*. *tumefaciens* genomic DNA with the primers BT493 and BT494 corresponding to the Atu5211open reading frames [14]. The PCR product was cloned into the cloning vector pGemT-Easy (Promega, USA) before the nucleotide sequence was determined. The *Apa*I-*Sac*I fragment was then subcloned into the broad-host-range plasmid pBBR1MCS-5 [34] digested with the same enzymes to yield pEstR.

The plasmid pEstR_C16S_ that expresses the mutant EstR_C16S_ in which Cys16 was exchanged to Ser, was constructed using PCR-based site-directed mutagenesis [7]. The mutagenic forward (BT3859) and reverse (BT3860) primers designed to change the Cys16 codon to Ser were used to amplify the pEstR plasmid. The PCR products were cut with *Apa*I-*Sac*I and ligated with similarly digested pBBR1MCS-5 [34]. The sequence of the mutated bases was verified using DNA sequencing.

The pEstC plasmid was constructed by amplification of the full-length *estC* gene (Atu5212) with the primers BT1559 and BT1595. The PCR product was cloned into pDrive, sequenced, and finally, the *Apa*I-*Pst*I fragment was subcloned into pBBR1MCS-3 [34] cut with the same enzymes to form pEstC.

The pEstC_S101A_ plasmid containing mutated EstC, in which Ser-63 was changed to Ala, was constructed using site-directed mutagenesis as described for the pEstR_C216S_ construction. The PCR products amplified from pEstC with the mutagenic forward (BT1663) and reverse (BT1662) primers were cut with *Apa*I-*Pst*I and ligated with similarly digested pBBR1MCS-3 [34].

### Construction of *estC*- and *estR*-promoter *lacZ* fusions

The *estC* and *estR* intergenic region, which contains putative *estC* and *estR* promoters located in opposite directions, was PCR amplified from NTL4 genomic DNA using the primers BT1574 and BT1575. The 314-bp PCR product was cloned into pGemT-Easy. The inserted DNA was sequenced to verify the correctness of the promoter. Then, an *Eco*RI fragment was cloned into *Eco*RI-cut pUFR047*lacZ* [4] containing a promoterless *lacZ*, yielding the pP_estC_ and pP_estR_ plasmids containing *estC* promoter-*lacZ* and *estR* promoter-*lacZ* fusions, respectively.

### Purification of EstC and EstR

The C-terminal His-tagged EstC protein was purified using the pETBlue-2 system (Novagen, Germany). An 810-bp full-length *estC* was amplified with primers BT1632 and BT1633 (see Table 1), and the *Nco*I-*Xho*I cut fragment was cloned into pETBlue-2 digested with the same enzymes, resulting in pETestC. *E*. *coli* BL21 (DE3) harboring pETestR was grown to exponential phase before being induced with 0.5 mM IPTG for 2 h. The bacteria were harvested and lysed by sonication. The clear lysate was loaded onto a Ni-NTA column (Qiagen, France). The EstC protein was eluted with a linear gradient of 0 to 100 mM imidazole.

To the purified, non-tagged EstR protein, a 483-bp PCR fragment containing the full-length *estR* amplified using the BT1621 and BT1622 primers was cut with *Nco*I and *Hin*dIII and cloned into the similarly digested pETBlue-2 (Novagen, Germany), yielding pETestR. An exponential phase culture of BL21 (DE3) harboring pETestR was induced with 0.5 mM IPTG for 18 h before being collected and lysed. Streptomycin sulfate (2.5% w/v) was added to the clear lysate to precipitate the nucleic acids prior to precipitating the protein with 70% saturated ammonium sulfate. The precipitated protein was resuspended in TED buffer (20 mM Tris pH 8.0, 1 mM EDTA pH 8.0, 0.1 mM phenylmethylsulfonyl fluoride, 2 mM dithiothreitol [DTT], 0.1 mM NaCl), loaded onto an Affi-Gel heparin column (Bio-Rad, USA), and washed extensively with TED buffer. Proteins were eluted from the column with a linear gradient of 0 to1.0 M NaCl. Fractions containing EstR were pooled and loaded onto a Q-Sepharose column (Amersham Bioscience, USA). Bound proteins were eluted a linear gradient of 0 to1.0 M NaCl. The eluted fraction containing EstR was dialyzed with TED buffer and concentrated by ultrafiltration (Amico Ultra-10K, Millipore, Germany). Purified protein was aliquoted and stored at -20°C. The purity of the purified protein was estimated from densitometric analysis of Coomassie blue stained gels after SDS-PAGE. Majority of the purified EstR presents in a reduced form as judged by nonreducing SDS-PAGE (data not shown).

### Gel mobility shift assay

BT1574 was 5’ end-labeled with [γ-^32^P] dATP using T4 polynucleotide kinase (Promega, USA). The [^32^P]-labeled *estR* and *estC* promoter fragments were amplified with [^32^P]-labeled BT1574 and BT1575 primers (see Table 1) and with pP_estC_ as the DNA template. Gel mobility shift reactions were performed in a 25 μl reaction mixture consisting of 3 fmol [^32^P]-labeled 314-bp PCR product, purified EstR (5–18 ng) and 1× binding buffer (20 mM Tris-HCl, pH 7.0, 50 mM KCl, 50 μg ml-1 BSA, 1 mM EDTA, 5% glycerol, 1 mM DTT). The binding was allowed to proceed at 25°C for 15 min. Protein-DNA complexes were separated by electrophoresis on 5% native polyacrylamide gels in Tris borate EDTA buffer (TBE) at 4°C.

### DNaseI foot printing

The DNaseI foot printing assay was performed in a 50 μl reaction mixture containing 1× binding buffer (20 mM Tris HCl, pH 7.0, 50 mM KCl, 1 mM EDTA, 5% glycerol, 50 μg ml^-1^ BSA, 5 μg ml^-1^ calf thymus DNA and 0.1 mM DTT), 500 ng poly(dI-dC), 20 ng [^32^P]-labeled *estR* and *estC* promoter fragments prepared as described for the gel mobility shift assay, and purified EstR at the indicated concentrations. The binding mixture was held at room temperature for 15 min. Fifty microliters of solution containing 5 M CaCl_2_ and 10 mM MgCl_2_ was added to the reaction prior to digesting it with 0.5 unit DNaseI for 30 s. Reactions were stopped by adding 700 μl stop solution (645 μl ethanol, 50 μl 3 M sodium acetate, and 5 μl 1 mg ml^-1^ yeast tRNA). The DNA was recovered by centrifugation for 15 min and resuspended in formamide loading buffer before being loaded onto 8% denaturing sequencing gels alongside the sequencing ladder created by PCR extension of the labeled M13F primer using pGem3ZF (+) as a template.

### Plate sensitivity assay

The resistance levels against oxidants were measured using plate sensitivity assays [6]. Serial dilutions of exponential phase cultures were made in LB broth, and 10 μl of each dilution was spotted on LB agar plates containing various oxidants, including CHP, BHP, MD, and H_2_O_2_. The plates were incubated at 30°C for 24 h before the colonies were counted. Experiments were performed in triplicate and the mean and standard deviation (SD) are shown.

### Enzymatic assays

Crude bacterial lysates were prepared, and protein assays were performed as previously described [35]. The total protein concentration in the cleared lysates was determined by a dye binding method (BioRad, USA) prior to their use in enzyme assays. β-galactosidase assays were performed using *o*-nitrophenyl-β-D-galactoside (ONPG) as a substrate, as previously described [36]. One international unit is the amount of enzyme generating 1 μmol of *o*-nitrophenol per min at 25°C [37]. Esterase activity was measured as previously described [21]. One esterase unit is defined as the amount of enzyme that liberates 1 μmol of *p*-nitrophenol per min at 25°C. Data shown are mean ± SD of triplicate experiments.

### Statistical analysis

The significance of differences between strains or cultured conditions was statistically determined using Student’s *t*-test. P < 0.05 is considered significant difference.

## Supporting Information

S1 Fig Purification of EstC. Purified EstC was separated by 15% SDS-PAGE. The gel was stained with coomassie blue. Lane 1, lysate from culture of BL21(DE3) harboring pETBlue-2 vector; Lane 2, lysate from uninduced culture of BL21(DE3) harboring pETestR; lane 3, lysate from IPTG-induced culture of BL21(DE3) harboring pETestR; lane 4 and 5, washing column fractions; lane 6, purified EstC.
(EPS)

S2 Fig Expression analysis of *estC*. Northern analysis of *estC* in *A*. *tumefaciens* NTL4 wild-type was performed using the [^32^P]-labeled *estC* probe and 20 μg of total RNA. UN, uninduced; CHP, induction with 1 mM CHP. The 23S rRNA used as the amount control is shown below the hybridized autoradiograph.
(EPS)

S3 Fig Purification of EstR. Purified EstR was separated by 15% SDS-PAGE. The gel was stained with coomassie blue. Lane 1, lysate from uninduced culture of BL21(DE3) harboring pETestR; lane 2, lysate from IPTG-induced culture of BL21(DE3) harboring pETestR; lane 3, purified EstR.
(EPS)
